# Deductive Reasoning Skills in Children Aged 4–8 Years Old

**DOI:** 10.3390/jintelligence12030033

**Published:** 2024-03-12

**Authors:** Krisztián Józsa, Tun Zaw Oo, Diana Borbélyová, Judit Podráczky

**Affiliations:** 1 Institute of Education, University of Szeged, 6722 Szeged, Hungary; 2 Institute of Education, Hungarian University of Agriculture and Life Sciences, 7400 Kaposvár, Hungary; oo.tun.zaw@uni-mate.hu (T.Z.O.); podraczky.judit@uni-mate.hu (J.P.); 3 MTA-MATE Early Childhood Research Group, Hungarian University of Agriculture and Life Sciences, 7400 Kaposvár, Hungary; borbelyovad@ujs.sk; 4 Department of Primary and Pre-School Education, J. Selye University, 94501 Komárno, Slovakia

**Keywords:** DIFER, deductive reasoning, young children, cross-cultural study, gender, family background

## Abstract

Young children possess the fundamental deductive reasoning skills for solving their upcoming problems in their daily lives. These skills are of great importance for their school readiness and academic development. Therefore, this study aimed to explore the age differences and predictive variables of deductive reasoning skills in young Hungarian children aged 4–8 years old who reside in Hungary and Slovakia. Face-to-face data were collected from 3050 participants. The instrument of deductive reasoning skills assessment was extracted from the school readiness test, DIFER (Diagnostic System for Assessing Development). Utilizing various statistical analyses with R, AMOS, and MPlus8 packages, it was found that there were significant differences in young children’s deductive reasoning skills across countries and age groups. Parents’ education levels had significant positive relationships with children’s deductive reasoning skills development. And the variables of country and age were identified as significant predictors of children’s deductive reasoning skills. And children’s family background variables such as parental education played a significant role in predicting children’s deductive reasoning skills in Hungary. The implications of this study emphasize the importance of educational contexts, parental involvement, cross-cultural exchange, and further research, with the potential to enhance young children’s educational experiences and prospects in Hungary, Slovakia, and beyond.

## 1. Introduction

Deductive reasoning, a fundamental cognitive skill, plays a pivotal role in shaping a child’s intellectual development. As children start the intricate process of learning and problem-solving, the ability to draw logical conclusions from given premises becomes increasingly vital (García-Madruga et al. 2022). When children tackle word problems, they depend on deductive reasoning skills to solve these challenges (Carreira et al. 2020). Under suitable conditions of support, elementary school children can exhibit fundamental deductive reasoning skills, and these abilities hold significant value for their upcoming challenges (de Chantal et al. 2020). Hence, the cultivation of deductive reasoning skills holds vital importance in the daily lives of children.

Deductive reasoning skills for children are the mental processes of striving to produce essential outcomes by starting from provided premises assumed to be accurate (de Chantal et al. 2020). Deductive reasoning is also simply defined by Solari (2014) as logical reasoning, the ability to reason or draw conclusions from the given premises. The acquisition of deductive reasoning skills in early childhood is a multifaceted and dynamic process, influenced by several factors. Age, as a central developmental marker, has a profound impact on a child’s cognitive growth (Heled et al. 2022). It is during these formative years that children embark on a quest to make sense of the world around them. Additionally, a child’s environment and family background can significantly shape their cognitive development. Parental education and socio-economic status are known to be crucial determinants in this regard (Demir-Lira et al. 2021). 

Young children’s general intelligence (g factor) plays a significant role in their cognitive skills progress (Almamari and Traynor 2021; Gomes et al. 2014; Panizzon et al. 2014). Moreover, language is central in the operations of children’s complex cognitive tasks, especially in deductive reasoning (Coetzee et al. 2023). It is also possible that the country of residence has a stronger influence on educational systems and teaching methods, which in turn affect deductive reasoning skills. Socio-economic status may have a more indirect or less pronounced effect on these cognitive skills within a specific cultural and educational context (Mohammad and Albaqawi 2023). Hence, this study explores the potential predictive role of family background variables on the progression of children’s deductive reasoning skills. This study also investigates differences in the development of deductive reasoning skills in young children across countries, genders, and age groups, focusing on those aged between 4 and 8 years in the captivating contexts of Hungary and Slovakia. 

## 2. Literature Review

### 2.1. Theoretical Perspectives on Deductive Reasoning

When discussing cognitive development and deductive reasoning, it is essential to address Piaget’s theory, as it plays a significant role in this context and cannot be overlooked. Piaget proposed that children progress at distinct cognitive stages, with deductive reasoning skills progressing through different age levels (Feiman et al. 2022; Evans 2005). As children advance through these stages, there might be age-related changes, and their thinking becomes more abstract and logical, impacting their deductive reasoning skills (Feiman et al. 2022). Therefore, age-related changes become an important factor to be considered in conducting research. 

Another influential perspective is the Information Processing Theory, which scrutinizes children’s deductive reasoning as a result of cognitive processes such as memory, attention, and problem-solving (Gagné 1993). This theory explores how children acquire, store, and manipulate information to draw logical conclusions. All children have individual differences based on their information-processing skills (Józsa et al. 2023a). Accordingly, individual differences including gender and age should be considered in researching children’s deductive reasoning skills assessment.

Social Cognitive Theory, developed by Albert Bandura, explores the role of social interactions and observational learning in cognitive development (Schunk and DiBenedetto 2020). According to this theory, children acquire deductive reasoning skills by observing others’ problem-solving and reasoning processes. Vygotsky’s Socio-Cultural Theory emphasizes the significance of social interactions and cultural context in cognitive development (Pathan et al. 2018). It posits that children’s deductive reasoning skills are shaped by their interactions with more knowledgeable individuals such as parents, teachers, and peers (Feiman et al. 2022). Therefore, it is crucial to consider the impacts of these knowledgeable individuals on children’s deductive reasoning skills development. In the field of educational psychology, researchers investigate the development of deductive reasoning skills within the context of formal education (Csapó et al. 2014; Józsa et al. 2023a; Nagy et al. 2004).

Together, these theoretical perspectives provide a multi-faceted understanding of the development of deductive reasoning skills in children, considering cognitive, social, cultural, and neuroscientific factors. These insights are invaluable for educators, psychologists, and researchers seeking to comprehend and support the growth of children’s deductive reasoning in various contexts. 

### 2.2. Deductive Reasoning in Early Childhood

The cognitive functioning of individuals undergoes rapid changes during early childhood, primarily due to the concurrent processes of brain development and the influence of environmental stimuli and experiences (D. Neumann et al. 2021). Early childhood, the formative years between 4 and 8 years, is a period marked by substantial cognitive, social, and emotional growth (Johnstone et al. 2022). At this stage, children begin to exhibit a range of cognitive abilities, including the capacity to engage in deductive reasoning (Simatwa 2010). Therefore, many researchers have assessed children’s deductive reasoning skills for their school readiness and further academic performance (Csapó et al. 2014; Józsa et al. 2023a).

Reasoning can be described as the capacity to draw conclusions, manipulate, and modify information. Deductive reasoning is a particular form of advanced cognitive skill that employs verbal, visual, or numeric premises to arrive at a rational outcome (Heled et al. 2022). For many decades, cognitive psychologists explored children’s deductive reasoning by asking them to generate logical conclusions based on provided premises (e.g., Franks 1997; Gazzo Castañeda et al. 2023; Markovits et al. 1989; Newton et al. 2010). Deductive reasoning in early childhood is characterized by the ability to draw logical conclusions from provided premises that are assumed to be true. It involves a systematic thought process wherein children follow a set of rules or principles to reach a specific and certain conclusion (Gazzo Castañeda et al. 2023). Deductive reasoning maintains the truth of statements, and its outcomes are restricted to being either true or false (Gazzo Castañeda et al. 2023). As they grow, their deductive reasoning skills become increasingly sophisticated, enabling them to make logical connections between information and form more complex deductions (Mohammad and Albaqawi 2023). 

Research in this area has shown that children, from early childhood on, begin to demonstrate rudimentary deductive abilities (de Chantal and Markovits 2017). For instance, they can solve basic logic puzzles and engage in simple deductive reasoning tasks. They show an understanding of concepts like “if-then” relationships, allowing them to infer conclusions based on given information (de Chantal and Markovits 2017; Józsa et al. 2023a; Nagy et al. 2004). Studies on deductive reasoning in early childhood have highlighted the importance of this cognitive skill for various aspects of a child’s development. For example, proficiency in deductive reasoning has been linked to improved problem-solving capabilities (Józsa et al. 2023a), mathematical understanding (Mohammad and Albaqawi 2023), and analytical reasoning skills (Carreira et al. 2020). Moreover, it plays a crucial role in academic achievement, particularly in subjects that require logical thinking and problem-solving, such as mathematics and science (Csapó et al. 2014). 

### 2.3. Deductive Reasoning, Insights from Mental Model Theory

The landscape of deductive reasoning, a complex facet of human cognition, has long been subject to scholarly exploration and debate. Apart from the above distinct theories attempting to shed light on this intricate cognitive process, Mental Model Theory (MMT) has emerged as a prominent and influential framework (Hattori 2016; Johnson-Laird and Byrne 1995). Over recent decades, MMT has played a pivotal role in shaping our understanding of reasoning processes, leaving an indelible mark on the cognitive and brain sciences (Barrouillet and Lecas 1998; Evans 1993; Gazzo Castañeda et al. 2023; Johnson-Laird and Byrne 1995; Mitchell 1993). In essence, MMT posits that humans possess a unique capacity to manipulate and represent information when engaged in reasoning and problem-solving activities. This capacity is made possible by the evolved visuospatial resources of the brain, allowing individuals to construct “mental models”—cognitive representations capturing essential information and spatial relationships among various elements (Cortes et al. 2023). Regarding the reasoning process, five primary principles of MMT have generally been the focus of research conducted by different researchers (Astorga 2016, 2017; Gauffroy and Barrouillet 2009); (1) the Principle of Representation, (2) the Principle of Inferences, (3) the Principle of Dual Systems, (4) the Principle of Modulation, and (5) the Principle of Verification. 

The first principle of MMT, the “Principle of Representation”, comes to life as individuals construct mental models during deductive reasoning tasks. Consider a statement logic scheme: “If I get a game, I am happy. Now, I got a game, so ------------- (I am happy).” In this example, a child, for instance, creates a mental representation linking the acquisition of a game to the feeling of happiness. The mental model, through abstraction, forms a spatial relationship within the cognitive framework, representing a crucial aspect of deductive reasoning (Astorga 2017; Gazzo Castañeda et al. 2023). 

As individuals construct these mental models, the second principle, the “Principle of Inferences”, takes center stage. In a predicate logic scheme like “The children are not adults yet. Tun is a child, so ---------- (not an adult yet)”, the spatial relationship within the mental model allows the child to infer that Tun is a child, is not an adult. The deductive leap from premises to conclusion relies on the mental model’s ability to encapsulate and organize information effectively (Astorga 2019; Evans 1993). 

The MMT also introduces the concept of two cognitive systems, giving rise to the third principle, the “Principle of Dual Systems”. This principle manifests the interplay between intuitive and analytical thinking during deductive reasoning (Gauffroy and Barrouillet 2009). For instance, consider a statement logic scheme: If it is a weekend, I play video games. But today is a weekday, so ------------ (I don’t play video games). In navigating this example, the child toggles between intuitive thinking linked to the routine of weekend gaming and analytical thinking to adapt to the spatial circumstances of a weekday. The dual systems, working in tandem, showcase the adaptive nature of deductive reasoning (Newton et al. 2010). 

Flexibility within deductive reasoning, a cornerstone of MMT, is supported by the fourth principle, the “Principle of Modulation”. In a statement logic scheme like “If I eat too much candy, I get a stomachache. However, today is a special occasion, so ----------- (I can eat some without getting a stomachache)”, the child modulates their mental model to accommodate the exceptional circumstance of a special occasion. This exemplifies how individuals can adjust their reasoning strategies based on contextual factors, focusing on the dynamic nature of deductive reasoning (Astorga 2016). 

The last principle, the “Principle of Verification”, rounds out MMT as individuals confirm the accuracy of their mental models. In a statement logic scheme such as “If I water the plants, they grow. Today, the plants are tall, so ------------ (I must have watered them)”, the child engages in cognitive verification, using observation and past experiences to affirm the validity of their deduction (Evans 2005). This principle underscores the self-checking mechanism inherent in deductive reasoning. 

The current study specifically concentrates on unraveling the intricacies of deductive reasoning through the lens of MMT, with a particular emphasis on two main principles, “Representation” and “Inferences”. By exploring these principles, this study aims to comprehend how children aged 4–8 construct mental models to represent information and draw inferences from given premises. These foundational aspects of MMT are crucial in investigating the cognitive processes underlying deductive reasoning in young children’s minds, shedding light on their evolving ability to create spatially organized mental models and make logical deductions based on these representations. 

### 2.4. Findings about Deductive Reasoning Skills

In the realm of elementary mathematics education, one study (Carreira et al. 2020) evaluates deductive reasoning through the introduction of a specific analytical reasoning problem. The finding showed that engaging children in reasonably intricate analytical reasoning exercises can assist in supporting a wide range of deductive reasoning development among young children. Teachers can foster the development of deductive reasoning abilities in young students by employing divergent thinking tasks, thereby improving their analytical and logical thought processes (García-Madruga et al. 2022). The authors evaluated students’ deductive reasoning abilities through a variety of training exercises. Deductive knowledge in this context encompasses several key principles: (a) ensuring that the statements and outcomes are in harmony; (b) recognizing that a valid conclusion is indispensable; (c) employing the effective strategy of seeking counterexamples as a means to arrive at a valid conclusion; and (d) emphasizing that, when seeking a necessary conclusion, reasoners should persist in evaluating all potential options, ensuring thorough and exhaustive work. Then, another study (de Chantal and Markovits 2017) examined the growth of deductive reasoning in preschool-aged children through the game, “Tell me if it’s Certain”. It involved four syllogistic reasoning challenges, each framed with a category-focused primary statement like “All As have B”. These problems permitted multiple potential starting points, such as “All dogs have legs”. The results indicate that the ability to generate alternative ideas is of greater significance than the capacity for inhibition in the context of logical reasoning among these young children. 

Regarding the relationship between parental education and deductive reasoning skills, one study (Demir-Lira et al. 2021) evaluated students’ deductive reasoning by the syllogistic reasoning tasks consisting of three premises and one conclusion. The results revealed that children with lower parental education displayed increased activity in the dorsolateral prefrontal regions of the brain. This occurred when they were confronted with set-inclusion and linear-order relation tasks, compared to their peers with more highly educated parents. These findings imply that children from less educated backgrounds may utilize spatial and cognitive control mechanisms to level the playing field and perform at a similar level to their counterparts with more educated parents. In the computer-based assessment of early reasoning and school readiness of first-grade children (438 participants) conducted by Csapó et al. (2014), gender differences were not found in children’s deductive reasoning skills development. Csapó et al. (2014) assessed students’ deductive reasoning through logical reasoning tasks. Each task began with two initial statements, and the children were required to draw a logical conclusion in the third sentence. 

Regarding age differences, Franks (1997) conducted experimental research to investigate age-related changes and the effect of age on children’s deductive reasoning skills. The finding showed a significant age effect on students’ reasoning tasks. In a study by Kaufman and colleagues (Kaufman et al. 2011), it was discovered that, at the level of individual age differences, performance on all reasoning tasks exhibited significant correlations and loaded onto a single factor representing deductive reasoning accuracy. There are many studies (Demetriou et al. 2008; Józsa et al. 2023a; Heled et al. 2022) highlighting the importance of age-related changes on students’ meta-cognitive development. Then, in another study about deductive reasoning in children’s school-readiness assessment in Hungary, the authors (Józsa et al. 2023a) applied the DIFER (Diagnostic System for Assessing Development) test. Their evaluation of deductive reasoning revealed a noteworthy and moderate association between children’s deductive reasoning abilities and their social skills, phoneme perception, relational reasoning, and early mathematics skills. However, the correlation between deductive reasoning and the development of fine motor skills was found to be weak. In this study, students’ reasoning skills development changed across different age groups of early childhood. 

An essential concept underlying individual differences is the empirically robust and pervasive nature of general intelligence, often referred to as “g” (Carroll 1993; Demetriou et al. 2023; Jensen 1998; Spearman 1927). General intelligence (g) holds a strong and well-established position in the field of psychology. Within psychometric theories of intelligence, “g” is considered a higher-level construct that arises from the interconnections among various cognitive tests, known as the “positive manifold” (Demetriou et al. 2008). Additionally, this factor showed a substantial correlation with “g”. The presence of “g” thus implies the potential existence of influential factors that impact performance on a wide array of complex cognitive tasks. 

In one study (Heled et al. 2022) investigating the relationship of children’s deductive reasoning skills with their working memory skills, its findings show that the deductive reasoning abilities of children can be anticipated based on their proficiency in working memory. In this study, students’ deductive reasoning assessment included two main tasks, such as the word context task and the deductive reasoning argument task. Another study (Markovits et al. 1989) investigated the development of elementary deductive reasoning skills in young children by using six logical syllogisms. It showed a distinct developmental trend, indicating that children’s capacity to distinguish their responses in relation to their deductive reasoning skills shows improvement throughout different age ranges (6, 8, and 11 years). In this study (Solari 2014), a deductive reasoning test known as the analysis–synthesis subtest from the Woodcock–Johnson III assessment was employed. This specific subtest assesses a dimension of fluid intelligence, concentrating on an individual’s capacity for deductive reasoning, which involves the ability to make reasoned judgments and draw conclusions based on provided information. The results of the study revealed a positive correlation between students’ deductive reasoning skills and their performance in mathematics.

After reviewing the findings concerning deductive reasoning skills mentioned above, it became evident that there are various tools available for assessing deductive reasoning. These include a proposed analytical reasoning problem, a range of training exercises, a game known as “Tell me if it’s certain”, syllogistic reasoning tasks, and the DIFER test. Additionally, the outcomes of these assessments vary depending on the specific goals of the research. Notably, factors such as students’ divergent thinking, working memory, general intelligence (g), social skills, parental education, socio-economic status, age groups, and gender differences all play a role in the development of children’s deductive reasoning skills. 

### 2.5. Context of the Study

To understand the context of the study, the educational backgrounds of both Hungary and Slovakia are briefly discussed. The Slovak Republic is a compact nation with approximately 5.5 million residents. At present, as many as 93% of the kindergartens in Slovakia are under public administration, receiving financial support from the government at both the national and local levels. Parents are responsible for a small financial contribution towards operational expenses, which includes covering meal costs. However, it is noteworthy that in the case of children in their final year of preschool, no fees are incurred, not even for meals (Pupala et al. 2022). As of 2020, parents with preschool-aged children in Slovakia join the collective of responsible parents, ensuring that their children are the subject of mandatory education, starting when the children are 5 years old (Majerčíková and Lorencová 2021). The present education structure in Slovakia is categorized into two primary segments: regional education and higher education, which encompasses universities. Regional education comprises institutions such as kindergartens, primary schools, secondary schools (including grammar schools, vocational schools, conservatories, and apprentice centers), specialized schools, and elementary art schools (Brečka et al. 2022). The Slovak curriculum is structured around criterion-oriented developmental support, enabling the definition and assessment of performance in relation to established requirements. This approach also allows for the characterization of cognitive skills at the achieved level (Podráczky et al. 2022; Zentai et al. 2023). 

Hungary (its population, around 9.8 million) has innovatively enhanced childcare and education for children aged 3–8 years old, implementing rigorous educator standards. The country has expanded opportunities and enhanced the learning environment for underprivileged children while also refining the assessment process (Józsa et al. 2018). Since 2016, the enrollment rate in Hungary has been 95% (Langer-Buchwald 2020). While the mandatory school age in Hungary starts at six, children are required to enroll in kindergarten as early as age three. The education system in Hungary places a strong emphasis on competition. The Hungarian education system explicitly incorporates competition as a legitimate objective in its curriculum design, assessment methods, and evaluation criteria (Nagy et al. 2018). The Hungarian core curriculum for preschool education is distinctive in its pluralistic, child-centered, and methodologically flexible approach. The Hungarian preschool curriculum places a strong emphasis on nurturing the child’s self-image and preschool experiences. It centers around engaging activities, especially games, and avoids rigid specifications. Instead, it relies on the developmental characteristics observed at the end of preschool as a guiding principle for educational advancement. 

Hungary and Slovakia, alongside Poland and the Czech Republic, are part of the Visegrád Group (V4). In this context, pre-primary and primary education play a crucial role as tools for shaping educational and social policies within the member states of the V4. As members of the V4, Slovakia and Hungary share not only geographical and historical connections but also cultural and historical roots, which naturally led to the development of similar educational systems (Kurincová et al. 2017). It is widely recognized that both countries construct their own educational systems. However, both countries share the responsibility of upholding common educational objectives and, in turn, contributing to the cultivation of competencies in children that will enable them to effectively function and thrive on an international scale (Borbélyová et al. 2019).

### 2.6. Rationale of the Study

After reviewing the theoretical perspectives on the development of deductive reasoning skills in children, several key considerations have emerged for further research in this area. Notably, Feiman et al.’s (2022) and Evans’s (2005) Piagetian viewpoints, as well as Gagné (1993) insights from Information Processing Theory, underscore the significance of factors such as children’s developmental stages, memory, attention, and problem-solving abilities in the development of deductive reasoning skills. Therefore, it is necessary to consider children’s individual developmental stages based on gender and age levels in assessing students’ deductive reasoning skills development. 

In addition, Bandura’s Social Cognitive Theory and Vygotsky’s Socio-Cultural Theory emphasize the role of observational learning and interactions with influential individuals like teachers, parents, and peers (Pathan et al. 2018; Schunk and DiBenedetto 2020). Accordingly, it is crucial to consider children’s socio-cultural backgrounds such as their family’s socio-economic status, their parents’ education levels, and the country or context in which they reside, in the assessment of children’s deductive reasoning skills. Drawing from these theoretical perspectives, our objective is to investigate individual variations in children’s development of deductive reasoning skills in relation to factors such as age, gender, and nationality. 

In alignment with our chosen theoretical foundation, Mental Model Theory (MMT), this study extends the current theoretical perspectives on deductive reasoning development. MMT, renowned for its emphasis on how individuals construct and manipulate mental representations during reasoning tasks, provides a useful lens to understand the intricacies of deductive reasoning in children (Johnson-Laird and Byrne 1995). By specifically focusing on the MMT principles of “Representation” and “Inferences”, the study aims to explore the spatially organized mental models children construct and the logical deductions drawn from provided premises. 

From the examination of the above-reviewed findings concerning deductive reasoning skills, it has become evident that activities involving analytical reasoning (Carreira et al. 2020), general intelligence (Demetriou et al. 2023), and logical processes (García-Madruga et al. 2022) contribute to the development of children’s deductive reasoning abilities. Additionally, children’s working memory (Heled et al. 2022) and their performance in mathematics (Solari 2014) display positive associations with the advancement of their deductive reasoning skills. Notably, significant disparities in children’s development of deductive reasoning skills are linked to their parents’ educational background and family circumstances (Demir-Lira et al. 2021). However, there were no noteworthy gender differences observed in the progression of children’s deductive reasoning abilities (Csapó et al. 2014). Based on these insights, a research gap has emerged in understanding the predictive influences of background variables and their dummy variables on children’s deductive reasoning skills development. Furthermore, there is a lack of studies exploring differences in children’s deductive reasoning skills development across age groups, genders, and countries. Therefore, our research objectives focus on examining the impacts of these background variables on the prediction of children’s deductive reasoning skills development. With these goals in mind, the following research questions were formulated for investigation. 

### 2.7. Research Questions

RQ1: Do children’s deductive reasoning skills exhibit significant differences with respect to their country, gender, and age groups?
RQ2: What are the significant differences between the two countries by gender and age?
RQ3: What is the relationship between children’s background characteristics and their deductive reasoning skills development?
RQ4: What are the factors that can predict the development of children’s deductive reasoning skills, considering their background characteristics?

## 3. Methods

### 3.1. Participants

Our study involved 3050 Hungarian children aged between 4 and 8 years, who were living in Hungary and Slovakia. Among these participants, 1441 were from Hungary, accounting for 47.25% of the total, and 1609 were from Slovakia, making up 52.80% of the total. Children who reside in Hungary are monolinguals who can speak only the Hungarian language, whereas those in Slovakia are bilinguals who can speak both Hungarian and Slovakian languages. Of these participants, 1641 were boys, comprising 53.82%, while the remaining 1409 children were girls, comprising 46.18%.

The sample was further categorized into various age groups, with 282 children at four years old (9.24%), 652 children at five years old (21.37%), 832 children at six years old (27.27%), 690 children at seven years old (22.62%), and 594 children at eight years old (19.48%). This study also asked for information about their family background situation, such as their mother’s education level, their father’s education level, and their socio-economic status. The detailed information is listed below (Table 1).

### 3.2. Instrument and Procedure

**Figure 1:**
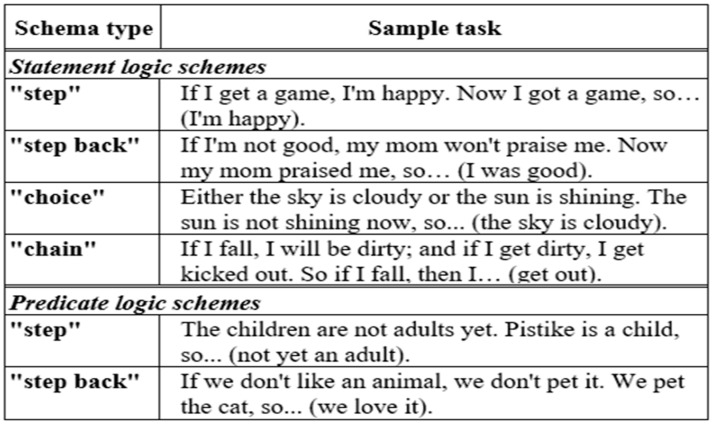
An example of a deductive reasoning task.

This tool for evaluating deductive reasoning skills in young children is a component of the DIFER (Diagnostic System for Assessing Development), a school readiness assessment used in Hungary and some Hungarian-speaking countries (Józsa et al. 2023a; Nagy et al. 2004). The deductive reasoning skills assessment utilized in the investigation corresponds to two key aspects of Mental Model Theory (MMT): the “Representation” principle and the “Inferences” principle. To assess children’s “Representation” skills, we gave this assessment item as an example, “*If I get a game, I am happy. Now, I got a game, so ------------- (I am happy)*”. Then, for the assessment of their “Inferences” skills, the sample item is “*The children are not adults yet, Pistike is a child, so --------- (not an adult yet)*”. An example of a deductive reasoning skills test is also described in the following figure (Figure 1). In our study, the deductive reasoning skills test comprises a set of 16 tasks designed to assess children’s deductive reasoning skills. The structure of the tasks is consistent across all schemes. Each task begins with two statements, referred to as premises (note that these may not always be two separate sentences in everyday language). The children are required to create the third statement, which is the conclusion, based on the information provided on the premises. Our aim is to present premises with content familiar to children, evoking situations most children can relate to. 

The tests were administered by trained BA in Education students in individual face-to-face sessions, taking an average of 10 min. During the standard examination, each child was assigned 16 tasks. The administrator sat across from the child to prevent them from viewing the answer key. Progressing through the tasks, the administrator read the two statements (premises) aloud, and the child was asked to complete the last, unfinished sentence (the conclusion). Each task was read once, with no repetition allowed. An example task sheet is described in Figure 1. 

The method for scoring responses remained consistent across all tasks. Each response was assessed with either a score of one (1) or zero (0), as per the instructions provided on the test sheet. The correct solution was indicated in the brackets beside each task, and evaluators simply determined whether the child’s answer aligned with the provided one in the bracket or not. It was essential to accept as correct (1) any answer that included elements of the correct conclusion with logical relevance, even if the child answered with additional elements. The results were assessed in two ways: (1) by calculating the children’s overall performances, ranging from 0 to 16 points; (2) by determining the children’s performance percentages such as preparatory level (0–29), beginner level (30–49), advanced level (50–69), finishing level (70–84), and optimum level (85–100).

### 3.3. Analysis

This current study harnessed various R packages, including stats, ggplot2, ggdist, and geom violin, and employed statistical techniques such as *t*-tests, ANOVA, correlational analyses by Chi-square, and different regression analyses (Escolano-Pérez and Bestué 2021). Furthermore, the study utilized AMOS version-23 for the measurement invariance of the deductive reasoning test and Mplus version 8.4 software for path analysis (Józsa et al. 2023b), and IBM SPSS Statistics 23.0 was employed for additional assessments for various descriptive statistics. To evaluate the goodness of fit of the model, recommended fit indices were relied upon. These recommended criteria included χ^2^/df < 5 for the Chi-square divided by degrees of freedom, RMSEA < 0.06 for the root mean square error of approximation, SRMR < 0.08 for the standardized root mean square residual, TLI > 0.90 for the Tucker–Lewis Index, and CFI > 0.90 for the comparative fit index, as outlined by Chua (2023) and Józsa et al. (2023a). Meeting these standards ensured that our model accurately represented the relationships among the variables and aligned with the observed data. For the measurement invariance of the test, researchers’ (Józsa et al. 2023a; Kline 2015) recommended values (∆CFI < 0.01, ∆RMSEA < 0.015, and ∆SRMR < 0.03) were referenced for metric, scalar, and residual models of the invariance assessment. In the investigation of the effects of categorical (dummy) variables, logistic regression analyses were conducted. In these analyses, this study also considered a result to be practically significant if the R2 value exceeded 0.13, following the guidelines outlined by Cohen (Mashar and Astuti 2022).

### 3.4. Reliability and Validity of the Instrument

The school readiness assessment, known as DIFER (which stands for “Diagnostic System for Assessing Development” in Hungarian), was validated for accuracy and consistency in a study (Józsa et al. 2023a). The instrument assessing the development of deductive reasoning skills in DIFER has previously been validated for its internal consistency reliability (Józsa et al. 2023a). In that study, a Cronbach’s alpha of 0.86, a composite reliability (CR) of 0.71, and an average variance extracted (AVE) of 0.50 were found, which attest to its reliability. The mean score for the deductive reasoning skills assessment was 66.77, with a standard deviation of 25.80. The instrument exhibits a normal distribution, as indicated by the acceptable skewness (−0.77) and kurtosis (−0.05) values. 

A review of the instrument’s developmental studies by Csapó et al. (2014); Józsa et al. (2023a); Nagy (1976); and Nagy et al. (2004) revealed that the assessment of deductive reasoning skills in the DIFER test is reliable and valid based on test–retest reliability and parallel-forms reliability measures. Test–retest reliability for the deductive reasoning assessment in the DIFER test ranged from r = 0.86 to 0.88, and the parallel-forms reliability was indicated by the correlation coefficient (r) = 0.73. Content validity is robust, with various versions implemented in different studies (Csapó et al. 2014; Józsa et al. 2023a; Lepes et al. 2016; Nagy 1976; Nagy et al. 2004).

This current study established the criterion validity of the DIFER, including the deductive reasoning skills assessment, in terms of concurrent and predictive evidence. For concurrent evidence, our DIFER, featuring the deductive reasoning skills assessment, has been used as a criterion-referenced test in Hungary, and it has undergone concurrent validation multiple times in different periods. Notably, it was integrated into the PREFER (Preventive Developmental Examination System for Children aged 4–7 years) test system in 1976, a project led by József Nagy and his colleagues. Concurrent validation has also been conducted by various renowned researchers (Csapó et al. 2014; Józsa et al. 2023a; Nagy 1976; Nagy et al. 2004). Consequently, concurrent validity is well established. For the predictive evidence, the DIFER test has been employed in several studies (Csapó et al. 2014; Józsa et al. 2018; Józsa et al. 2023a) to predict children’s school readiness and academic development in future studies. Thus, the criterion validity of the instrument in our study has been substantiated. 

### 3.5. Confirmatory Factor Analysis and Baseline Models of the Instrument

**Figure 2:**
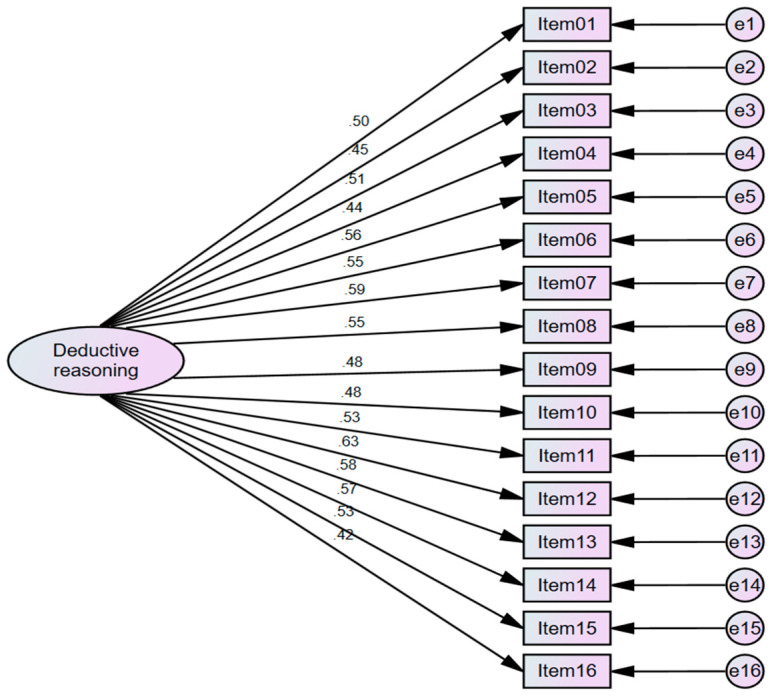
CFA model for deductive reasoning skills assessment (N = 3050).

To investigate the measurement invariance (MI) of the instrument, multi-group confirmatory factor analysis (MG-CFA) was employed for comparing different groups. Initially, a confirmatory factor analysis (CFA) model was executed to validate a theoretical model postulating relationships between observed variables and underlying latent factors. The model demonstrated favorable fit indices: Chi square = 593.27, df = 104, CFI = 0.953, TLI = 0.934, RMSEA = 0.039, SRMR = 0.028 (Figure 2). Factor loadings, indicating item–factor correlations, showed an acceptable range (>0.40) (Józsa et al. 2023a). Therefore, the CFA model of the instrument was confirmed.

Subsequently, baseline models were constructed to evaluate the MI of deductive reasoning skills across distinct country groups, genders, and age groups. The established models (as shown in Table 2) align with the recommended fit indices (Józsa et al. 2023a). The presented fit indices affirm the satisfactory goodness of fit of the baseline models, thus facilitating the examination of the MI across diverse demographic groups. 

### 3.6. Measurement Invariance of the Deductive Reasoning Test

Measurement invariance of the deductive reasoning test was explored by multi-group confirmatory factor analysis (MG-CFA). In the MG-CFA of country levels (Hungary and Slovakia), all measurement models, such as configural, metric, scalar, and residual, are accepted as they are consistent with the recommended MI cut-off scores. Specifically, the configural model was assessed and demonstrated a strong model fit. Subsequently, metric invariance was examined by constraining factor loadings to be equal for Hungarian-speaking children in both countries. The comparison between configural and metric models showed no significant decrease in fit, indicating full invariance of factor loadings across countries (∆CFI = 0.001, ∆RMSEA = −0.001, and ∆SRMR = 0.001). In the analysis of scalar invariance, item intercepts were constrained to be the same across groups. The results also demonstrated that the fit of the model did not significantly decrease in the scalar model (∆CFI = −0.001, ∆RMSEA = −0.001, and ∆SRMR = 0.000). To assess residual invariance, item residuals were constrained. The fit indices supported the adequacy of this residual model (∆CFI = −0.007, ∆RMSEA = 0.001, and ∆SRMR = 0.002), indicating residual invariance across countries. These findings align with the recommended thresholds for metric, scalar, and residual invariance (∆CFI < 0.01, ∆RMSEA < 0.015, and ∆SRMR < 0.03) as outlined by researchers Józsa et al. (2023a) and Kline (2015). Therefore, this indicates that the overall measurement invariance of the deductive reasoning test between Hungary and Slovakia was maintained. 

At the gender level, the relationships in the deductive reasoning test demonstrated strong model fits across various models, including configural, metric, scalar, and residual. When comparing the configural and metric models, they met the predetermined fit index thresholds (∆CFI = 0.001, ∆RMSEA = −0.001, and ∆SRMR = 0.000). And there was also no significant decrease in fit between the metric and scalar models (∆CFI = 0.000, ∆RMSEA = −0.001, and ∆SRMR = 0.000). Additionally, the fit indices of the residual invariance model did not significantly differ from those of the scalar invariance model (∆CFI = 0.001, ∆RMSEA = −0.001, and ∆SRMR = 0.001), as shown in Table 3. These results indicate that the deductive reasoning test maintains measurement invariance across genders, providing support for its reliability and validity in assessing children’s deductive reasoning skills development. 

At the age level, the measurement invariance of the deductive reasoning test was investigated across different age groups (4th year, 5th year, 6th year, 7th year, and 8th year). Both configural and metric models demonstrated measurement invariance in the structural and factor loadings of the deductive reasoning test across all age groups, with acceptable fit indices (∆CFI = −0.006, ∆RMSEA = 0.000, and ∆SRMR = 0.006). The scalar invariance was established across different age groups with acceptable model fit indices (∆CFI = −0.004, ∆RMSEA = 0.008, and ∆SRMR = 0.018). However, the deductive reasoning test did not meet the criteria for residual invariance with recommended values (∆CFI = −0.110, ∆RMSEA = 0.008, and ∆SRMR = 0.014). Despite the lack of residual invariance at the age level, the deductive reasoning test has invariances of configural, metric, and scalar models. Therefore, the deductive reasoning test demonstrates a valid and acceptable measurement invariance across age groups in the study (Goñi et al. 2020; Kim et al. 2022).

### 3.7. Latent Mean Differences across Groups

After establishing full scalar measurement invariances of the deductive reasoning test for country, gender, and age groups, the group mean differences of latent variables were explored (Chiu et al. 2015; Goñi et al. 2020; Kim et al. 2022). Slovakia served as the reference group in the country-level comparison, while boys were used as the reference in the gender-level analysis. For age group comparisons, the 4th year was the reference group in contrast to other age groups, the 5th year for comparisons with senior age groups, the 6th year for comparisons with other senior age groups, and the 7th year for comparisons with the 8th year (Table 4). No significant differences were observed in comparisons of latent means in countries and genders. This means that children from both countries exhibit comparable levels of latent abilities, and so do genders. However, significant differences were found across all age groups in their latent abilities, favoring older age groups. The possible reason may be that according to Piaget’s theory of cognitive development, children increase their cognitive skills based on their developmental states by age. However, it is interesting to know how they are different in practice, by assessing their deductive reasoning skills development.

## 4. Results

### 4.1. Addressing RQ1: Investigating the Differences in Children’s Deductive Reasoning Skills across Countries, Genders, and Age Groups

#### 4.1.1. Differences between the Two Countries

**Figure 3:**
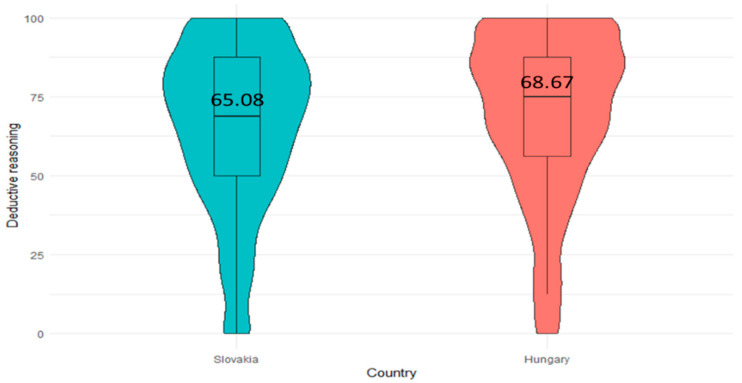
Comparison of deductive reasoning skills between countries (N_Slovakia_ = 1609, N_Hungary_ = 1441).

To examine variations in deductive reasoning skills among children concerning their country of origin, this study employed a *t*-test using the R packages of “stats” and “ggplot2”. Our analysis revealed a significant difference (*** *p* < 0.001) between these two countries, Hungary (M = 68.67, SD = 25.76) and Slovakia (M = 65.08, SD = 25.73). However, the effect size (Cohen’s d = 0.14) is low, and thus, students’ deductive reasoning skills development is almost the same in level. The reason for the significant difference may be attributed to the large sample size of our study. Figure 3 further illustrates these distinctions between the two countries, providing a clear visual representation of the comparison in deductive reasoning skills observed in the study. 

#### 4.1.2. Differences between Genders

**Figure 4:**
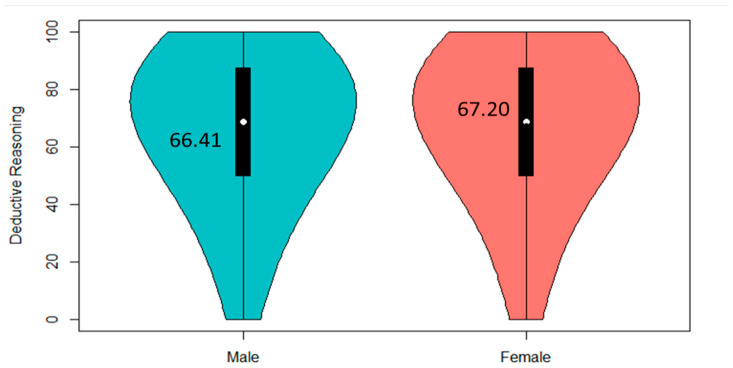
Comparison of deductive reasoning skills between genders (N_male_ = 1641, N_female_ = 1409).

Then, the study also explored the examination of variations in deductive reasoning skills among children based on their gender. The study utilized the *t*-test in R for this analysis, and the result indicated that there was no statistically significant difference (*p* > 0.05, Cohen’s d = 0.03) between male children (M = 66.41, SD = 25.87) and female children (M = 67.20, SD = 25.72). The comparison between genders is also visually represented in Figure 4, providing a graphical depiction of the lack of significant differences in deductive reasoning skills between male and female children. This suggests that, in the context of our study, gender may not appear to be a significant factor influencing deductive reasoning skills development. 

**Figure 5:**
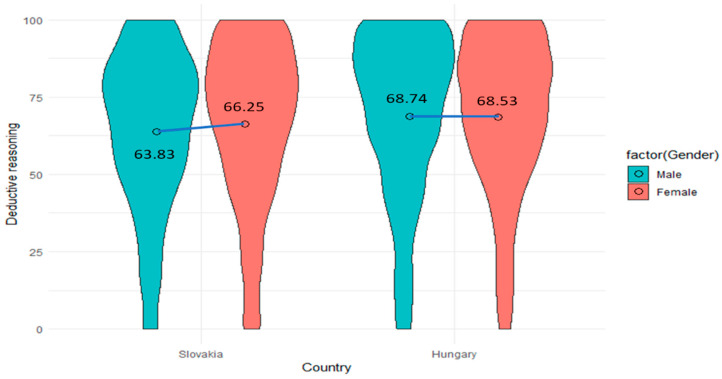
Comparison of deductive reasoning skills between genders for both countries (N_male/Slovakia_ = 779, N_female/Slovakia_ = 830; N_male/Hungary_ = 862, N_female/Hungary_ = 579).

For each country, independent samples *t*-tests were conducted to investigate the gender differences in children’s deductive reasoning skills development. In Slovakia, the investigation showed that there was no statistically significant difference (*p* > 0.05) between boys and girls. Similarly, in Hungary, no significance was found in differences between genders (*p* > 0.05). These results are visually presented in Figure 5 for enhanced clarity. One of the possible reasons may be that the educational systems in both countries might not favor one gender over the other in terms of deductive reasoning skills development.

#### 4.1.3. Differences across Age Groups

**Figure 6:**
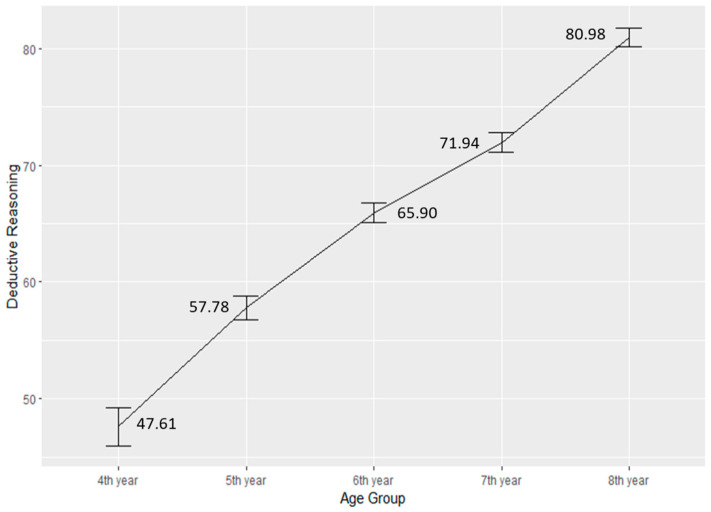
Comparison of deductive reasoning skills by the age groups (N4th _year_ = 282. N5th _year_ = 652, N6th _year_ = 832, N7th _year_ = 690, N8th _year_ = 594).

When comparing children’s deductive reasoning skills across five different age groups (4th year, 5th year, 6th year, 7th year, and 8th year), this study employed a one-way ANOVA analysis using R. The results indicated significant differences among the age groups (F (4, 3045) = 129.50; *** *p* < 0.001) regarding their development of deductive reasoning skills. Specifically, children in the 4th year displayed the lowest level of development in deductive reasoning skills (M = 47.61, SD = 27.77). Subsequently, there was an ascending trend in deductive reasoning skills development with the 5th year (M = 57.78, SD = 26.40), 6th year (M = 65.90, SD = 24.55), 7th year (M = 71.94, SD = 22.21), and 8th year (M = 80.98, SD = 19.40) age groups (Figure 6). This finding is consistent with Piaget’s cognitive development theory highlighting that the cognitive development of children by age contributes to their deductive reasoning skills development. This information is valuable for understanding the developmental trajectory of deductive reasoning skills in children as they advance through their education. Figure 5 visually depicts these differences among the age groups. 

**Figure 7:**
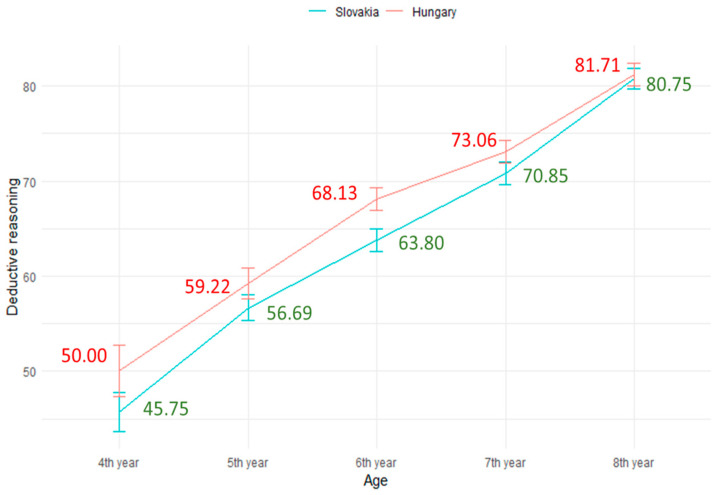
Comparison of deductive reasoning skills by the age groups (N4th _year (Hungary,Slovakia)_ = 123, 159. N5th _year (Hungary,Slovakia)_ = 282, 370, N6th _year (Hungary/Slovakia)_ = 403, 429, N7th _year (Hungary,Slovakia)_ = 339, 351, N8th _year (Hungary,Slovakia)_ = 294, 300).

This study also investigated the age differences in children’s deductive reasoning skills development in both countries. Utilizing ANOVA, the results revealed substantial variations across five different age groups (F (4, 1436) = 52.00; *** *p* < 0.001) in Hungary. Children of older ages developed their deductive reasoning skills more than younger ones. Similarly, in Slovakia, significant differences were observed among different age groups (F (4, 1604) = 76.98; *** *p* < 0.001). Older children developed more deductive reasoning skills compared to their younger peers. One possible explanation for this trend could be that deductive reasoning skills tend to significantly improve at later ages (Feiman et al. 2022). The results are visually presented in Figure 7. 

### 4.2. Addressing RQ2: Comparison of Two Countries by Gender and Age Groups

**Figure 8:**
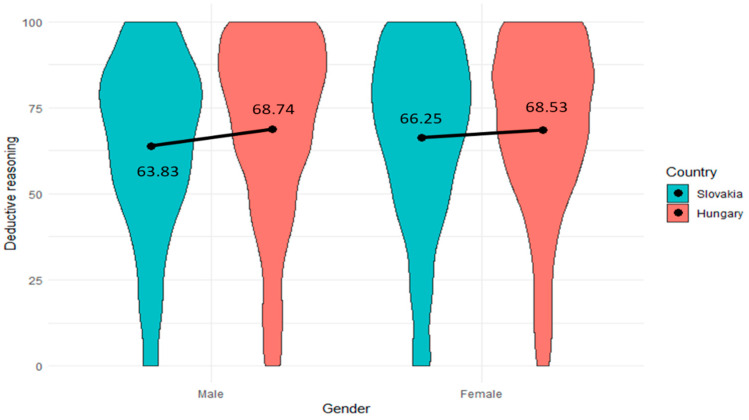
Comparison of deductive reasoning skills between two countries by gender (N_male (Slovakia, Hungary)_ = 779, 862; N_female (Slovakia, Hungary)_ = 830, 579).

The study conducted a gender-based comparison of children’s deductive reasoning skills development in two countries, Hungary and Slovakia. To compare boys in Hungary and Slovakia, the *t*-test was utilized. The results indicated a significant difference (*p* < 0.001, Cohen’s d = 0.19), with schoolboys from Hungary (M = 68.74, SD = 26.20) showing more advanced deductive reasoning skills than those from Slovakia (M = 63.83, SD = 25.27). However, when comparing girls, no significant difference (*p* > 0.05) was observed between the two countries. The development of schoolgirls’ deductive reasoning skills was almost the same in both countries (Figure 8). The lack of a significant difference in girls’ performance between the two countries suggests that gender-based distinctions in deductive reasoning skills development do not appear to be present among this particular group of children.

**Figure 9:**
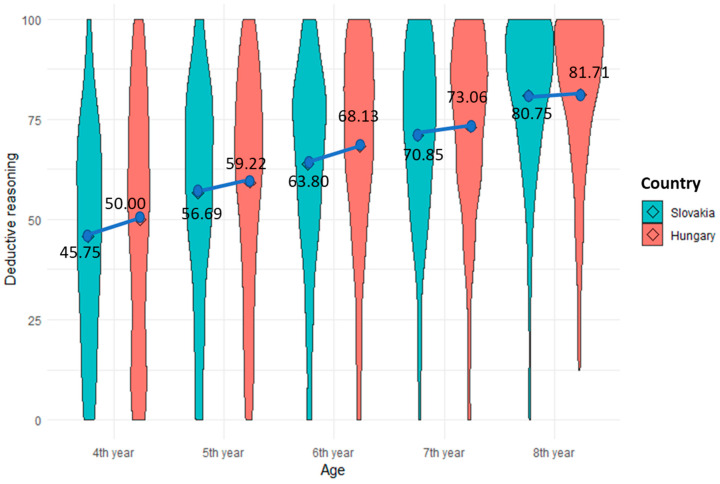
Comparison of deductive reasoning skills between countries by the age groups (N4th _year (Hungary,Slovakia)_ = 123, 159, N5th _year (Hungary,Slovakia)_ = 282, 370, N6th _year (Hungary/Slovakia)_ = 403, 429, N7th _year (Hungary,Slovakia)_ = 339, 351, N8th _year (Hungary,Slovakia)_ = 294, 300).

To compare the development of children’s deductive reasoning skills in different age groups (4th year, 5th year, 6th year, 7th year, and 8th year) between the two countries, the study used independent samples *t*-tests. Firstly, when comparing the 4th-year age group in terms of deductive reasoning skills between the two countries, no significant difference was found (*p* > 0.05). Similarly, for other age groups (5th year, 7th year, and 8th year), no significant differences were observed between the countries for these age groups, except for the 6th-year age group. Notably, when comparing the 6th-year age group, a significant difference in children’s deductive reasoning skills development between the two countries emerged (Cohen’s d = 0.17). Children from Hungary exhibited greater development in deductive reasoning skills (M = 68.13, SD = 24.42, *p* < 0.05) than those from Slovakia (M = 63.80, SD = 24.52). For clarity, a visual presentation is shown in Figure 9. The reason may be that cultural norms, language usability (monolingual or bilingual), and parental involvement can impact a child’s educational progress, and these factors may have been more influential for the 6th-year age group in Hungary than in Slovakia. 

### 4.3. Addressing RQ3: Relationship between Children’s Background Variables and Deductive Reasoning Skills

The current study explored how various background factors of children, including the country they reside in, gender, parental education, and socio-economic status, relate to their development of deductive reasoning skills across different age groups (4th year, 5th year, 6th year, 7th year, and 8th year). Among the five age groups, the study identified significant relationships in the 7th-year and 8th-year age groups concerning parental education and the development of deductive reasoning skills. In the 7th-year age group, it was seen that the level of education attained by mothers (r = 0.161, *p* < 0.01) and fathers (r = 0.131, *p* < 0.01) had substantial positive correlations with children’s deductive reasoning skills development. Similarly, in the 8th-year age group, it was found that the mother’s education level (r = 0.128, *p* < 0.01) and father’s education level (r = 0.105, *p* < 0.01) also displayed noteworthy positive associations with the development of children’s deductive reasoning skills (Table 5). The reason may be that educated parents may serve as role models for their children, emphasizing the importance of education and deductive reasoning skills development. 

### 4.4. Addressing RQ4: Predicting Effects of Background Variables on Deductive Reasoning Skills

**Figure 10:**
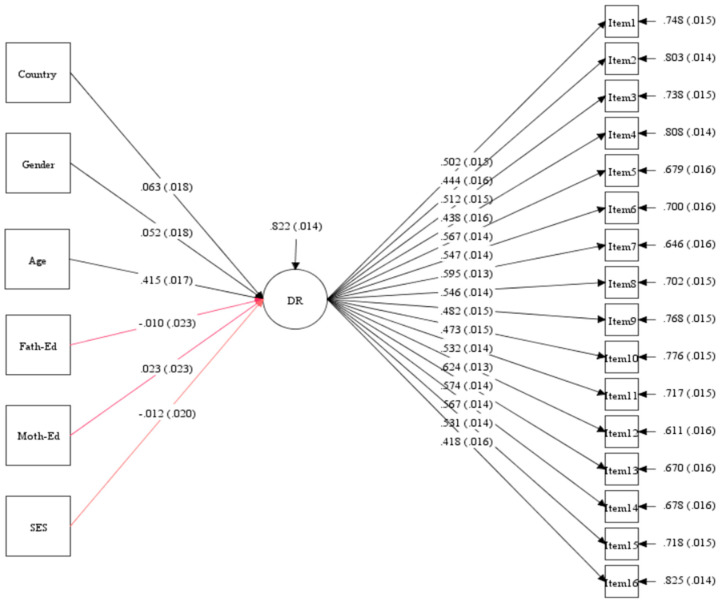
The prediction model of background variables on deductive reasoning skills development (N = 3050).

To examine how children’s background factors (country, gender, age, mother’s education, father’s education, and socio-economic status) influence the development of their deductive reasoning skills, the study utilized Mplus8 software for our analysis. The initial focus was on assessing the suitability of the prediction model and its fitness for the data. The model demonstrated a strong fit, as evidenced by favorable fit indices: Chi square = 729.80, df = 194, *p* < 0.001. Additionally, the model displayed a comparative fit index (CFI) of 0.95, Tucker–Lewis Index (TLI) of 0.945, standardized root mean square residual (SRMR) of 0.02, and root mean square error of approximation (RMSEA) of 0.03. Furthermore, the coefficient determination (R^2^ = 0.822) was also greater than the recommended value (0.13 by Mashar and Astuti 2022); it was found that the model was well fitted (as shown in Figure 10). 

Upon evaluating the model, it was evident that children’s age had the most pronounced and statistically significant impact on the development of their deductive reasoning skills. In contrast, factors such as the country in which they reside and their gender had statistically significant but comparatively weaker effects on their deductive reasoning skills. Notably, variables related to family background, such as parental education and socio-economic status, did not exhibit a significant influence on the development of children’s deductive reasoning skills. 

In addition to examining the predictive impacts of individual background variables mentioned above, the study conducted a series of logistic regression analyses aimed at gaining a comprehensive understanding of how categorical variables (dummy variables) influence the development of children’s deductive reasoning skills (Table 6). When exploring the effects of different countries, it was observed that Hungary had a stronger predictive influence (β = 0.069, *p* < 0.001) on children’s deductive reasoning skills development compared to the referenced variable, Slovakia. In the investigation of age-related dummy variables (with the 4th year as the reference category), all age groups, namely the 5th, 6th, 7th, and 8th years (β = 0.162, β = 0.316, β = 0.395, β = 0.512, *p* < 0.001) demonstrated more significant predictive effects compared to the 4th-year age group on children’s deductive reasoning skills development. Furthermore, when examining the socio-economic factors, it was found that families with average economic status had a more substantial effect (β = 0.093) than low-income families in predicting children’s deductive reasoning skills development. 

### 4.5. Predicting Effects of Background Variables for Each Country

For the individual countries, the regression models were constructed by MPlus23 to investigate the predicting effects of background variables such as gender, age, parental education, and SES on children’s deductive reasoning skills development. Regarding the data from both Hungary and Slovakia, the predicting models first showed good model fits (R^2^ _Hungary, Slovakia_ = 0.85, 0.77) with the recommended fit indices, as shown in Table 7. 

**Figure 11:**
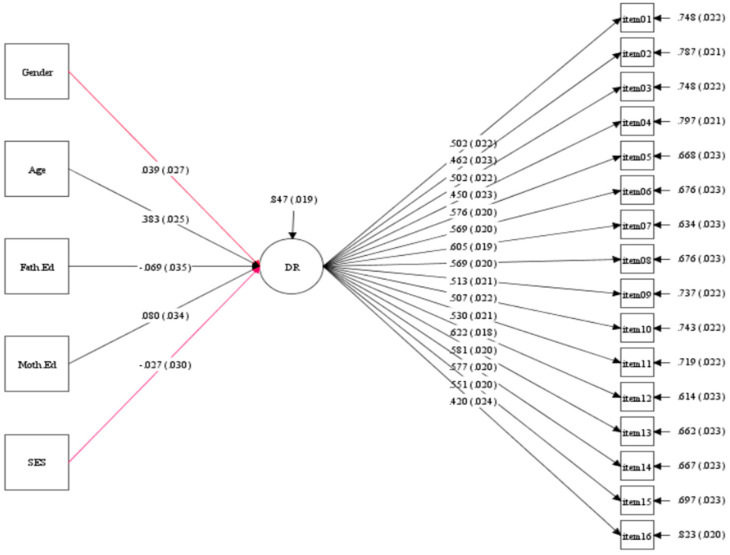
The prediction model of background variables on deductive reasoning skills development in Hungary (N = 1441).

In the predictive model concerning children from Hungary, it was observed that certain background variables, such as age and parental education, had noteworthy effects on the development of children’s deductive reasoning skills (as illustrated in Figure 11). The age variable exerted a significant and moderately influential effect (β = 0.383, *p* < 0.001) on children’s deductive reasoning skills. Similarly, variables like the mother’s education level (β = 0.080, *p* < 0.05), and the father’s education level (β = −0.069, *p* < 0.05) demonstrated significant impacts on children’s deductive reasoning skills development. The reason may be that the age-related improvement in deductive reasoning skills can be attributed to cognitive development and the accumulation of knowledge over time. The mother’s education level likely positively influences deductive reasoning through a supportive learning environment and role modeling. The slightly negative impact of the father’s education level may be influenced by socio-economic or cultural factors that affect parental involvement in a child’s education. 

**Figure 12:**
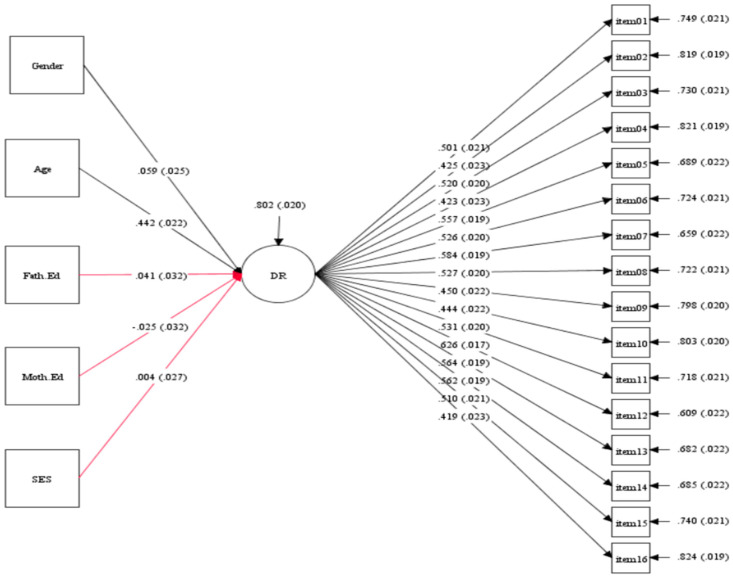
The prediction model of background variables effects on deductive reasoning skills development in Slovakia (N = 1609).

In the examination of the predictive model for children from Slovakia, it was found that both gender and age significantly influenced the development of deductive reasoning skills. More precisely, a child’s age demonstrated a notable and moderately impactful effect (β = 0.442, *p* < 0.001) on deductive reasoning skills, whereas gender had a weak significant influence (β = 0.059, *p* < 0.001). The model depicting these relationships is presented in Figure 12. The reason may be that the improvement in deductive reasoning skills with age among children is likely due to cognitive development and the accumulation of knowledge and experience over time. Gender differences in deductive reasoning may stem from societal influences and expectations, which can shape the way children approach deductive reasoning tasks.

The study investigated both factors, examining the impact of dummy variables related to various background factors on the development of children’s deductive reasoning skills through logistic regression analyses (Table 8). When studying the influence of different age groups on deductive reasoning skills in Hungary, it was found that the older age groups, namely the 5th year, 6th year, 7th year, and 8th year, had more substantial predictive effects (β = 0.142, 0.316, 0.380, and 0.488, *p* < 0.001) compared to the 4th-year age group. Furthermore, among the educational levels of mothers, the level of tertiary education had a more significant predictive effect (β = 0.076, *p* < 0.05) than other education levels. Finally, the average SES of families in Hungary exhibited more pronounced effects compared to other SES levels on the development of children’s deductive reasoning skills. Similarly, in Slovakia, it was observed that the older age groups, including the 5th year, 6th year, 7th year, and 8th year, exhibited more significant predicting effects (β = 0.179, 0.310, 0.403, and 0.530, *p* < 0.001) than the 4th-year age group on children’s deductive reasoning skills development. Moreover, when it comes to the SES of families, findings showed that families with a tertiary SES level had a more significant predictive effect (β = 0.079) than families with lower SES levels in shaping children’s deductive reasoning skills. The reason for these results may be attributed to the fact that as children grow older, their deductive reasoning skills tend to improve, which is reflected in the increasing predictive effects of older age groups. Additionally, the level of the mother’s education, particularly tertiary education, may play a more substantial role in shaping a child’s deductive reasoning skills. Finally, the average SES of families may have a stronger impact as it represents a more comprehensive measure of the overall environment and resources available to children for skills development.

## 5. Discussion

This research aimed to investigate the development of deductive reasoning skills in children while considering various background variables. To evaluate the findings, the study examined how they relate to the research questions and objectives, explored their implications, and identified studies that align with our research findings. The MI of the deductive reasoning test and latent mean differences of cross-cultural groups were first confirmed and investigated for the reliability of the findings from this research.

One of our primary research questions aimed to investigate whether children’s deductive reasoning skills exhibit significant differences based on their country of residence. The findings reveal a slight variation in children’s deductive reasoning skills between Hungary and Slovakia. Although education systems and children’s language possessions (monolinguals or bilinguals) may contribute to slight differences, the effect size of the difference between the two countries is low. Therefore, it can be assumed that no distinct difference was found between the children of the two countries, regarding the deductive reasoning skills development. Research by Evans (2005) and Feiman et al. (2022) provides theoretical perspectives on cognitive development, which can be considered in explaining these disciplines. Piagetian viewpoints highlight the significance of cognitive stages in children’s development, which may impact minor differences between countries due to variations in educational systems and cultural influences. 

Then, the study investigated the potential variations in deductive reasoning skills between boys and girls. Following Feiman et al.’s (2022) and Evans’s (2005) Piagetian viewpoints, there might be differences in individual development in boys or girls. Our findings, however, indicated that there were no statistically significant differences in deductive reasoning skills based on gender. It is important to examine the broader literature to understand why this may be the case. Csapó et al.’s (2014) research is in line with our finding, indicating that gender may not be a significant factor in deductive reasoning skills development. This suggests that cognitive development in this domain may be relatively gender-neutral, and other factors like age, culture, or educational practices may play a more critical role.

This study also explored the role of age in the development of deductive reasoning skills. Our results revealed a clear developmental trend, with older children exhibiting more advanced deductive reasoning abilities. This is consistent with the cognitive development literature. The theoretical perspectives discussed in our research background, such as Piaget’s cognitive stages and Information Processing Theory, support this finding. These theories highlight the maturation of cognitive abilities and the acquisition of problem-solving and logical thinking skills with age. The ascending trend in deductive reasoning skills observed in our study mirrors this theoretical understanding. To further align our findings with previous research, studies on cognitive development in children, such as those conducted by Franks (1997), Heled et al. (2022), and Solari (2014), emphasize the role of age differences and their association with cognitive development, such as deductive reasoning. The development of deductive reasoning skills is intricately tied to cognitive skills, and older children are likely to have more developed deductive reasoning skills. 

One of the notable findings from our research was the positive correlation between parental education and the development of deductive reasoning skills, particularly in the 7th- and 8th-year age groups. These findings align with previous research (Demir-Lira et al. 2021; Kaufman et al. 2011) and suggest that educated parents can serve as influential role models for their children, emphasizing the importance of parental education in deductive reasoning skills development. This underscores the critical role that parents play in nurturing their children’s cognitive abilities. When exploring the impact of different education levels (primary, secondary, and tertiary), it was observed that the level of tertiary education had a more significant predictive effect on children’s deductive reasoning skills compared to primary education. 

Another focus of our study was the examination of family background variables and their impact on deductive reasoning skills. When analyzing the entire sample, the study identified that the country of the children’s residence and their age levels played significant predicting roles in the development of deductive reasoning skills. The above distinct theories, such as Piaget’s cognitive development theory, Bandura’s Social Cognitive Theory and Vygotsky’s Socio-Cultural Theory, offer insights into the role of age-related changes, social interactions, and observational learning in cognitive development. These theories imply that differences in social and cultural practices within these countries and age-related changes might impact children’s deductive reasoning. 

In the specific examination of each country, it was seen that parental education and children’s age in Hungary had significant predicting effects on their deductive reasoning skills development. This finding aligns with one distinct study (Demir-Lira et al. 2021), supporting the notion that family circumstances and socio-economic status can significantly influence children’s deductive reasoning skills. In the case of Slovakia, it was evident that a child’s age had a more substantial impact on their deductive reasoning skills development. Although the overall socio-economic status (SES) could not predict children’s deductive reasoning skills in either country, the average SES level (when analyzing the categorical variables) exhibited more significant predicting effects compared to other SES levels. This could be attributed to the members of the V4 countries, in which Hungary and Slovakia share not only geographical and historical connections but also cultural and historical roots, leading to the development of similar education systems (Kurincová et al. 2017). Future research could explore how family background variables, such as SES, might influence other cognitive domains or the development of deductive reasoning skills. 

It is essential to consider these findings when designing educational interventions and policies, especially for children from diverse socio-economic backgrounds. Further research in this field could explore the specific mechanisms through which family background variables moderate the relationship between country and deductive reasoning skills, providing a deeper understanding of these complex dynamics. Additionally, investigating whether there are any subgroups within age or gender categories that exhibit different patterns of deductive reasoning skills development could shed more light on these relationships. It is worth noting that while our findings align with studies emphasizing the role of family background in cognitive development (Mohammad and Albaqawi 2023), there may be research that suggests a more direct influence of gender on the deductive reasoning skills development of children. Therefore, ongoing research should continue to explore different factors that contribute to the development of deductive reasoning skills in children and how these factors may interact to produce the observed outcomes. Furthermore, general intelligence (g) can be different in different age groups, which might have some effects on children’s deductive reasoning skills development. Therefore, future research is recommended to investigate the influence of the “g” factor on children’s deductive reasoning skills development. 

Despite the valuable insights gained from this research, it is important to acknowledge some limitations of this study. First, the study focused on deductive reasoning skills, but cognitive development is a multifaceted process, and there might be age-related changes. Future research should consider a broader range of cognitive abilities, potentially including general intelligence for different ages, working memory, inductive reasoning, critical thinking, and problem-solving skills, to provide a more comprehensive picture of cognitive development. Second, the findings of the study may be influenced by various unexamined variables, such as teaching methods, educational curricula, general intelligence (g factor) for different ages, or cultural factors, which were not included in the analysis. Investigating these factors could lead to a more nuanced understanding of the observed differences in deductive reasoning skills. Third, the study focused on a specific sample of 3050 participants, and, thus, it has a limitation in generalizing the findings to the comparison of entire countries (Hungary and Slovakia). Finally, the study evaluated the current development of children’s deductive skills; however, it did not employ longitudinal data to specifically track developmental changes. As a result, it is recommended that future research address these gaps. 

## 6. Conclusions

In conclusion, this research contributes to our understanding of deductive reasoning skills development in children, highlighting variables such as country, age, and family background factors. No significant differences were found in children’s deductive reasoning skills across countries and genders. Age emerged as a significant predictor of deductive reasoning skills development, with older children demonstrating more advanced skills. Family background variables, specifically parental education, also play a role in predicting children’s deductive reasoning skills development in Hungary. While gender did not significantly impact deductive reasoning skills in our study, it is important to continue exploring the potential influence of other variables not examined in this research. 

These findings have practical implications for educators and policymakers, highlighting the importance of tailored educational strategies that consider the diverse backgrounds and developmental stages of children. The intricate relationship between country, age, and family background encourages the need for nuanced interventions to support children’s cognitive development. Overall, this study emphasizes the complex nature of deductive reasoning skills development and the diverse factors that contribute to this cognitive process, providing a foundation for further research and informed educational strategies. 

## Figures and Tables

**Table 1:** Descriptive statistics of the study.

Variables	Number	Percent
*Country*	(Total—3050)	-
Slovakia	1609	52.80%
Hungary	1441	47.25%
*Gender*	*(2 groups)*	-
Male	1641	53.82%
Female	1409	46.18%
*Age*	*(5 groups)*	-
4th year	282	9.24%
5th year	652	21.37%
6th year	832	27.27%
7th year	690	22.62%
8th year	594	19.48%
*Mother’s education*	*(3 groups)*	-
Primary	522	17.08%
Secondary	1692	55.47%
Tertiary	836	27.41%
*Father’s education*	*(3 groups)*	-
Primary	497	16.3%
Secondary	1948	63.9%
Tertiary	605	19.8%
*Socio-economic status*	*(3 groups)*	-
Low	458	15.02%
Average	2307	75.44%
High	285	9.34%

**Table 2:** Baseline models for countries, genders, and age groups.

Models	Chi Square/df	*p*	CFI (≥0.90) *	TLI (≥0.90) *	RMSEA [90% CI] (≤0.07) *	SRMR (≤0.07) *
Hungary	341.31/104	<0.001	0.954	0.947	0.040 [0.035, 0.045]	0.030
Slovakia	402.78/104	<0.001	0.942	0.933	0.042 [0.038, 0.047]	0.032
Male	300.66/104	<0.001	0.964	0.959	0.034 [0.030, 0.038]	0.027
Female	442.84/104	<0.001	0.931	0.920	0.048 [0.044, 0.053]	0.036
4th year	202.02/104	<0.001	0.905	0.901	0.058 [0.046, 0.070]	0.051
5th year	256.59/104	<0.001	0.926	0.915	0.047 [0.040, 0.055]	0.041
6th year	224.69/104	<0.001	0.948	0.940	0.037 [0.031, 0.044]	0.034
7th year	204.30/104	<0.001	0.939	0.930	0.037 [0.030, 0.045]	0.037
8th year	229.00/104	<0.001	0.915	0.901	0.045 [0.037, 0.053]	0.041

Note: * (acceptable value).

**Table 3:** Measurement invariance across countries, genders, and age groups.

Models	Chi Square/df	CFI	RMSEA	SRMR	∆CFI (<0.01) *	∆RMSEA (<0.015) *	∆SRMR (<0.03) *	Decision
*Country (Hungary, Slovakia)*								
Configural	744.10/208	0.948	0.029	0.032	-	-	-	Accept
Metric	753.70/223	0.949	0.028	0.033	0.001	−0.001	0.001	Accept
Scalar	774.50/238	0.948	0.027	0.033	−0.001	−0.001	0.000	Accept
Residual	864.80/254	0.941	0.028	0.035	−0.007	0.001	0.002	Accept
*Gender (Male, Female)*								
Configural	743.50/208	0.948	0.029	0.027	-	-	-	Accept
Metric	752.40/223	0.949	0.028	0.027	0.001	−0.001	0.000	Accept
Scalar	762.20/238	0.949	0.027	0.027	0.000	−0.001	0.000	Accept
Residual	773.70/254	0.950	0.026	0.028	0.001	−0.001	0.001	Accept
*Age (4th year, 5th year, 6th year, 7th year, 8th year)*								
Configural	1116.80/520	0.930	0.019	0.051	-	-	-	Accept
Metric	1227.60/580	0.924	0.019	0.057	−0.006	0.000	0.006	Accept
Scalar	1365.60/640	0.915	0.020	0.058	−0.009	0.001	0.001	Accept
Residual	2366.80/704	0.805	0.028	0.072	−0.110	0.008	0.014	Reject

Note: * (recommended value).

**Table 4:** Group mean differences in latent variables.

Groups	Mean Difference (MD)	Standard Error (SE)	Critical Ratios (CR)	Effect Size (d)
Slovakia (Reference) vs. Hungary	0.03	0.005	12.583	0.141
Boys (Reference) vs. girls	0.01	0.004	12.821	0.046
4th year (Reference) vs. 5th year	0.09	0.006	10.743 *	0.350
4th year (Reference) vs. 6th year	0.16	0.004	11.286 ***	0.821
4th year (Reference) vs. 7th year	0.22	0.003	10.671 ***	0.899
4th year (Reference) vs. 8th year	0.29	0.002	10.002 ***	0.978
5th year (Reference) vs. 6th year	0.10	0.004	11.270 ***	0.418
5th year (Reference) vs. 7th year	0.15	0.003	10.659 ***	0.851
5th year (Reference) vs. 8th year	0.23	0.002	9.992 ***	0.936
6th year (Reference) vs. 7th year	0.10	0.003	10.640 ***	0.389
6th year (Reference) vs. 8th year	0.18	0.002	9.977 ***	0.912
7th year (Reference) vs. 8th year	0.14	0.002	9.962 ***	0.800

Note. * (*p* < 0.05), *** (*p* < 0.001), effect size (d) was calculated by the formula d = [k2]/ϕ^½^, where [k2] is the mean difference of latent groups, and ϕ is the estimated variance score assumed to be homogeneous for both latent groups (Cohen 1988; Hancock 2001).

**Table 5:** Correlational analyses among background variables and deductive reasoning skills.

4th Year	2	3	4	5	6
1. Country 2. Gender 3. Mother’s ed. 4. Father’s ed. 5. SES 6. Deductive reasoning	0.020	0.072 0.090	0.009 0.073 0.588 **	0.005 −0.007 0.437 ** 0.317 **	0.076 −0.075 0.026 0.066 0.070
5th year	2	3	4	5	6
1. Country 2. Gender 3. Mother’s ed. 4. Father’s ed. 5. SES 6. Deductive reasoning	−0.124 **	−0.016 0.024	−0.004 0.061 0.668 **	0.031 0.088 0.430 ** 0.419 *	0.048 0.055 0.014 0.023 0.009
6th year	2	3	4	5	6
1. Country 2. Gender 3. Mother’s ed. 4. Father’s ed. 5. SES 6. Deductive reasoning	−0.069 *	−0.010 0.058	−0.002 −0.010 0.563 **	−0.002 −0.028 0.297 ** 0.243 **	0.088 * 0.063 0.031 0.041 −0.015
7th year	2	3	4	5	6
1. Country 2. Gender 3. Mother’s ed. 4. Father’s ed. 5. SES 6. Deductive reasoning	−0.126 **	−0.025 0.035	0.002 0.065 0.634 **	0.045 0.040 0.488 ** 0.499 **	0.050 0.084 0.161 ** 0.131 ** −0.004
8th year	2	3	4	5	6
1. Country 2. Gender 3. Mother’s ed. 4. Father’s ed. 5. SES 6. Deductive reasoning	−0.206 **	−0.012 0.016	0.008 −0.015 0.642 **	0.081 * −0.029 0.329 ** 0.337 **	0.012 0.034 0.128 ** 0.105 ** −0.070

Note. * (*p* < 0.05), ** (*p* < 0.01)

**Table 6:** Regression analyses for the predictions of dummy variables for the whole sample.

Model	Dummy Variables	Unstandardized Coefficients		Standardized Coefficients	t	Sig	Correlations			Collinearity Statistics	
		Β	Std. Error	β			Zero-Order	Partial	Part	Tolerance	VIF
*Country* (Slovakia/reference)	Hungary	3.58	0.934	0.069	3.843	<0.001	0.069	0.069	0.069	1.000	1.000
*Gender* (Boy/reference)	Girl	0.794	0.937	0.015	0.195	0.195	0.015	0.023	0.023	0.987	1.013
*Age * (4th year/reference)	5th year	10.177	1.701	0.162	5.982	<0.001	−0.182	0.108	0.100	0.384	2.604
	6th year	18.289	1.645	0.316	11.119	<0.001	−0.021	0.198	0.186	0.348	2.873
	7th year	24.332	1.687	0.395	14.422	<0.001	0108	0.253	0.242	0.375	2.667
	8th year	33.370	1.726	0.512	19.331	<0.001	0.271	0.338	0.324	0.400	2.501
*Mother’s ed* (Primary/reference)	Secondary	−1.152	1.292	−0.022	−0.892	0.373	0.001	−0.016	−0.016	0.530	1.888
	Tertiary	−1.949	1.440	−0.034	−1.35	0.176	−0.018	−0.025	−0.025	0.530	1.888
*Father’s ed * (Primary/reference)	Secondary	−0.953	1.297	−0.018	−0.735	0.462	−0.013	−0.013	−0.013	0.563	1.777
	Tertiary	−0.486	1.563	−0.008	−0.331	0.756	0.004	−0.006	−0.006	0.563	1.777
*SES * (Low/reference)	Average High	5.569	1.316	0.093	4.231	<0.001	−0.076	−0.076	−0.076	0.680	1.471
		−2.637	1.941	−0.030	−1.358	0.174	0.023	−0.025	−0.025	0.680	1.471

Note: Dependent variable is the deductive reasoning skills.

**Table 7:** Model fit indices for each country.

Model	Chi-Square/df	*p*-Value	SRMR (≤0.07) *	CFI (≥0.9) *	TLI (≥0.9) *	RMSEA [90% CI] (≤0.06) *
Hungary	414.101/179	<0.001	0.027	0.956	0.951	0.030 [0.026, 0.034]
Slovakia	490.674/179	<0.001	0.028	0.943	0.936	0.033 [0.029, 0.036]

Note: * (recommended values).

**Table 8:** Regression analyses for the predictions of dummy variables for each country.

Model	Dummy Variables	Hungary			Slovakia		
		β	Std. Error	*p*-Value	β	Std. Error	*p*-Value
*Gender* (Boy/reference)	Girl	−0.003	1.385	0.903	0.047	1.283	0.060
*Age * (4th year/reference)	5th year 6th year 7th year 8th year	0.142 0.316 0.380 0.488	2.605 2.484 2.538 2.589	<0.001 <0.001 <0.001 <0.001	0.179 0.310 0.403 0.530	2.238 2.191 2.256 2.315	<0.001 <0.001 <0.001 <0.001
*Mother’s ed* (Primary/reference)	Secondary Tertiary	−0.051 0.076	1.871 2.098	0.175 <0.05	0.003 0.005	1.778 1.970	0.928 0.882
*Father’s ed* (Primary/reference)	Secondary Tertiary	−0.004 0.015	1.865 2.239	0.919 0.673	−0.028 −0.028	1.798 2.173	0.396 0.403
*SES * (Low/reference)	Average High	0.088 −0.043	1.932 2.723	<0.01 0.189	0.097 −0.022	1.791 2.774	<0.01 0.457

Note: Dependent variable is the deductive reasoning skills.

## Data Availability

Data are available upon appropriate request.
